# Effect of Vitamin D3 on the Levels of Oxidative Stress and Expression of the NLRP3 Inflammatory Gene in Type 2 Diabetes Mellitus: A Randomized Clinical Trial

**DOI:** 10.1002/hsr2.70770

**Published:** 2025-04-29

**Authors:** Shadi Behshad, Yaser Mohammadi, Gholamreza Anani Sarab, Mohammad Malekaneh, Azam Rezaei Farimani

**Affiliations:** ^1^ Department of Biochemistry, Faculty of Medicine Birjand University of Medical Sciences Birjand Iran; ^2^ Student Research Committee Iran University of Medical Sciences Tehran Iran; ^3^ Department of Biochemistry, School of Medicine Iran University of Medical Sciences Tehran Iran; ^4^ Medical Toxicology and Drug Abuse Research Center (MTDRC) Birjand University of Medical Sciences Birjand Iran

**Keywords:** diabetes, inflammation, NLRP3, oxidative stress, randomized clinical trial, vitamin D3

## Abstract

**Background and Aims:**

Type 2 diabetes mellitus (T2DM) is a chronic metabolic disorder characterized by insulin resistance and chronic inflammation. The activation of the NLRP3 inflammasome is a key contributor to the inflammatory processes associated with T2DM, which can exacerbate disease progression. This study aims to evaluate the effects of vitamin D3 supplementation on oxidative stress markers and NLRP3 gene expression in patients with T2DM.

**Methods:**

Sixty‐eight patients with T2DM, exhibiting HbA1c levels greater than 6.5% and serum 25(OH) vitamin D3 levels below 30 ng/mL, were enrolled in this randomized controlled trial. Participants were assigned to either a vitamin D3 group (*n* = 34), receiving 50,000 IU/week for 8 weeks, or a placebo group (*n* = 34). Serum levels of oxidative stress markers (malondialdehyde [MDA], total antioxidant capacity [TAC], and thiol levels) and NLRP3 gene expression were assessed at baseline and after the intervention.

**Results:**

The vitamin D3 group demonstrated a significant increase in serum 25(OH) vitamin D3 levels compared to the placebo group (*p* < 0.001). However, no significant changes were observed in oxidative stress markers (MDA, TAC, and thiol levels) between the groups. Importantly, NLRP3 gene expression was significantly reduced in the vitamin D3 group compared to the placebo group (*p* < 0.02).

**Conclusion:**

These findings suggest that vitamin D3 supplementation may effectively reduce inflammation in T2DM patients by lowering NLRP3 expression. This supports the potential role of vitamin D3 as an adjunctive therapy for managing inflammation and oxidative stress in individuals with T2DM.

## 1 Introduction

Type 2 diabetes mellitus (T2DM) is a chronic metabolic disorder characterized by insulin insensitivity as a result of insulin resistance, decreased insulin production, and ultimately pancreatic beta‐cell insufficiency [1]. The disease is spreading worldwide with an increasing prevalence, thus it is expected that 366 million individuals get this disease by 2030, of whom 77.6% belong to developing countries [2].

The abnormal activity of the innate immune system in metabolic disorders such as T2DM is known as an important mechanism of disease pathogenesis [3]. The emergence of a chronic pro‐inflammatory state due to the activation of innate myeloid immune cells is directly related to the emergence of insulin resistance [3, 4]. According to the previous evidence, the inflammasome, as the cytosolic multi‐protein molecular platform in myeloid cells, can sense which control the secretion of pro‐inflammatory cytokines in metabolic stress [5]. The inflammasome structurally consists of a NOD‐like receptor (NLR), an apoptosis‐related spot‐like protein containing CARD adapter protein, and caspase‐1{Nucleotide‐binding and oligomerization domain} [5, 6]. One type of the NLR molecule is a NLRP3 [6].

NLRP3 gene (known as CIAS1) is located on the long arm of chromosome 1 [7] and forms the central nucleus in the NLRP3 inflammasome that is involved in the inflammation process [8]. There is an impression that the regulation and activation of the NLRP3 inflammasome occurs at both transcriptional and posttranslational levels [9]. Accordingly, the upregulation of the expression of NLRP3 is the first signal in inflammasome activation induced by the toll‐like receptor/nuclear factor NF‐κB pathway. The secondary signal is transduced by various pathogen and damage‐associated molecular patterns and activates the NLRP3 inflammasome to form a multiprotein complex assembly [10] This complex, in turn, catalyzes the conversion of procaspase‐1 to caspase‐1 and subsequently induces the production and secretion of inflammatory cytokines interleukin (IL)‐1β and IL‐18 [9, 11].

Despite the existence of a variety of danger signals known for NLRP3, reactive oxygen species (ROS) appears to be a common danger signal for NLRP3 inflammasome expression and activation [12, 13] Oxidative stress (OS) increases in diabetic patients with glycaemic poor control or insulin resistance and is caused by abnormal metabolisms such as hyperglycemia, dyslipidemia, and elevated levels of free fatty acids [14] Recent research has highlighted the link between NLRP3 inflammasome, OS, and IL‐1β production during metabolic stress, providing a new approach to research and treatment of T2DM [15].

25 OH Vitamin D3 (VD3) and its active form, namely, 1, 25 (OH) 2 VD3 are necessary for human physiological functions such as reducing inflammation and excessive intracellular OS [16]. Therefore, VD3 deficiency increases the incidence and severity of several age‐correlated metabolic disorders interrelated with OS. Additionally, VD3 is a potent antioxidant that improves mitochondrial activity while preventing OS‐related protein oxidation, lipid peroxidation (LOP), and DNA damage [17] In this regard, VD3 supplementation has been proposed as a potential intervention to lower diabetes risk and its complications [18]Thus, the present study was conducted to investigate the effect of VD3 on oxidative stress markers and NLRP3 gene expression in patients with T2DM.

## 2 Methods

### 2.1 Trial Design

This study was a randomized, double‐blind, placebo‐controlled clinical trial conducted at the Diabetes Clinic in South Khorasan, Birjand, from November 2019 to February 2020. The trial adhered to the principles outlined in the Declaration of Helsinki and was approved by the Ethics Committee of Birjand University of Medical Sciences with the ethics code IR.BUMS.REC.1398.140. It was also registered on the IRCT website under the code IRCT20200616047795N1. Written informed consent was obtained from all participants before the commencement of the study.

### 2.2 Participants

A total of 68 patients, aged 50‐65 years, with T2DM and vitamin D deficiency (25(OH)VD3 levels < 30 ng/mL) participated. Eligible patients had at least 5 years of diabetes history, with HbA1c > 6.5. Exclusion criteria included recent VD3 supplementation (within the last 3 months), concurrent diseases (e.g., cancer, thyroid, kidney, adrenal, or liver diseases), corticosteroid use, and any change in medication type or dosage during the study period.

### 2.3 Sample Size

Given multiple outcome measures, the sample size was calculated based on the malondialdehyde (MDA) variable: *α* = 0.05, *β* = 0.2, with an effect size of 20% and an attrition rate of 20%. This yielded a final sample size of 34 patients per group using the clinical trial formula with a 1:1 group ratio [19].

### 2.4 Intervention and Procedures

Participants were randomly allocated to either the intervention or placebo group using a finite block randomization method (block size = 4) stratified by age (two groups: > 50 years) and gender (*n* = 34). In the intervention group, patients received an oral dose of 50,000 IU of vitamin D3 (VD3) weekly for 8 weeks, while the control group received identical placebo capsules. To ensure blinding, both VD3 and placebo capsules manufactured by Zahavi (Tabriz, Iran) were identical in appearance and packaging, and neither participants nor research staff were aware of the group allocations. Participants were instructed to maintain their regular physical activity and avoid any additional nutritional supplements for the duration of the study.

### 2.5 Outcomes

The primary outcome of this study was the change in serum VD3 levels. Secondary outcomes included the expression levels of the NLRP3 inflammatory gene and oxidative stress markers, specifically serum malondialdehyde (MDA), total antioxidant capacity (TAC), and thiol group levels.

### 2.6 Laboratory Testing

At baseline and after 8 weeks, blood samples (10 mL) were collected from each participant following a 10‐h fasting period. Each sample was divided into two 5 mL aliquots. One aliquot was used to measure vitamin D3 levels and oxidative stress markers after centrifugation at 2500 rpm for 10–15 min. The oxidative stress markers, including malondialdehyde (MDA), total antioxidant capacity (TAC), and thiol levels, were measured according to the manufacturer's instructions (Zantox, Iran). The second aliquot was processed to isolate peripheral blood mononuclear cells (PBMCs) for gene expression analysis. Vitamin D3 levels were quantified using a chemiluminescence assay.

RNA was extracted from PBMCs using a commercial RNA extraction kit (Yektatajhiz, Iran), followed by DNase treatment to prevent genomic DNA contamination. cDNA synthesis and quantitative real‐time PCR (qRT‐PCR) for NLRP3 and the housekeeping gene GAPDH were performed according to manufacturer protocols (Yektatajhiz, Iran). Expression data were analyzed with the 2^−^^ΔΔCt^ method to quantify relative changes.

### 2.7 Statistical Analysis

Statistical analyses were performed using SPSS software (Version 16). The distribution of data was assessed using the Shapiro–Wilk test. Gender differences between groups were analyzed with the Pearson Chi‐square test. For within‐group comparisons (pre and postintervention), paired *t*‐tests were used for parametric variables, while Wilcoxon tests were applied for nonparametric variables. Between‐group comparisons were conducted using independent *t*‐tests or the Mann‐Whitney test, as appropriate. A *p* value of less than 0.05 was considered statistically significant. Additionally, GraphPad software was utilized to visualize changes in gene expression. To further assess potential interactions between time and intervention effects, a two‐way repeated measures ANOVA was conducted for key biochemical markers, including BMI, blood pressure, HbA1c, TAC, MDA, and thiol levels.

## 3 Results

### 3.1 Participants

**Figure 1:**
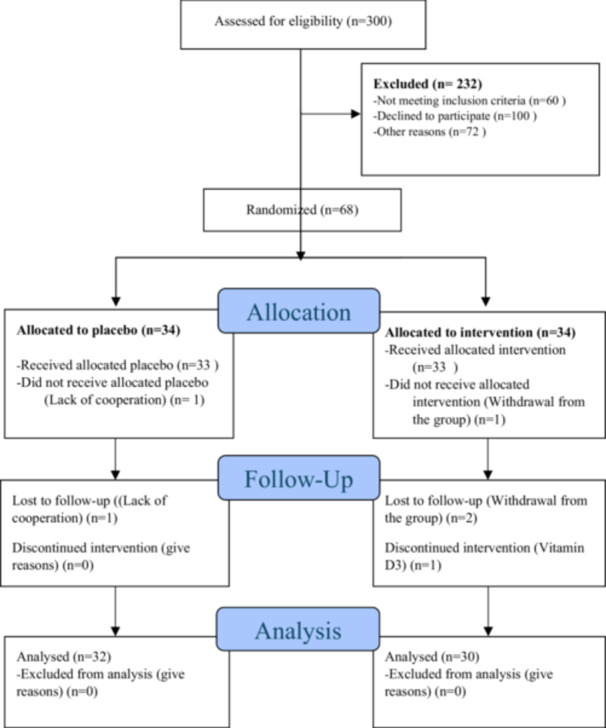
CONSORT diagram for the study (subject participation, randomization, follow‐up, and analysis).

Among the 68 patients in the allocation phase, one participant from both the intervention and placebo groups was excluded due to a lack of cooperation. Additionally, four participants (two from each group) withdrew during the follow‐up phase. Consequently, 62 participants completed the study: 30 in the VD3 group and 32 in the placebo group. Figure 1 illustrates the allocation and follow‐up process.

### 3.2 Baseline Characteristics

Table 1 presents the baseline characteristics of the participants. Among the 62 controlled diabetic patients, 59.7% (*n* = 37) were male, and 40.3% (*n* = 25) were female. Additionally, there was no significant difference in gender distribution between the two groups (*p* = 0.96).

**Table 1:** Baseline characteristics of patients.

Variable	Vitamin D3 group (*n* = 30)	Placebo group (*n* = 32)	*p* value (difference between groups)
Age (years)	57.30 ± 4.750	57.22 ± 5.302	0.95**
Male sex (%)	60% (18/37)	59.4% (19/37)	0.96*
Female sex (%)	40% (12/25)	40.6% (13/25)	
Duration of diabetes (years)	9.27 ± 3.25	8.88 ± 3.44	0.65*

* *p* value is reported based Chi‐Square test

** *p* value is reported based on the analysis of independent‐sample *t*‐test.

### 3.3 Effect of VD3 on Biochemical Parameters

At the beginning of the study, there was no significant difference in the serum levels of VD3 and oxidative stress markers between the two groups. After 8 weeks, the results were as follows: The serum level of VD3 had a significant increase in the VD3 group compared to the beginning of the study (*p* < 0.001, Table 2).

**Table 2:** Comparative changes of the anthropometric and biochemical parameters in the Vitamin D3 and placebo groups after 8 weeks of vitamin D3 supplementation.

variables	placebo group (*n* = 32)			Vitamin D3 group (*n* = 30)			*p* value end‐of‐trial (difference between groups)**
	Baseline	End‐of‐trial	*p* value*	Baseline	End‐of‐trial	*p* value*	
Body mass index (BMI)	25 ± 3	25 ± 3	0.14	26 ± 3	25.60 ± 3	0.52	0.62
Systolic BP (mmHg)	134 ± 19	131 ± 15	0.23	135 ± 18	134 ± 18	0.92	0.51
Diastolic BP (mmHg)	73 ± 9	72 ± 8	0.60	73 ± 1	71 ± 8	0.37	0.74
25(OH) VD3 (ng/ml)	19 ± 7	20 ± 8	0.37	18 ± 6	40 ± 1	0.0001	0.0001
HbA1c	7.91 ± 1.93	7.94 ± 1.48	0.64	8.29 ± 1.66	7.79 ± 1.52	0.12	0.70
TAC (µmol/l)	560 ± 103	540 ± 87	0.13	555 ± 124	541 ± 105	0.44	0.96
MDA (µmol/l)	3 ± 2	4 ± 3	0.20	3 ± 2	3 ± 2	0.14	0.62
Thiol Group (µmol/l)	349 ± 44	342 ± 52	0.45	343 ± 67	351 ± 73	0.44	0.90

* *p* values for comparing baseline values with the end line within each group (Time‐dependent). A paired sample *t*‐test was used for parametric variables and Wilcoxon for nonparametric variables

** *p* values for comparing the changes of variables between the groups (VD3 vs. Placebo). An independent‐sample *t*‐test was used for the parametric variables, and the Mann‐Whitney test was used for the nonparametric ones.

No significant difference was found in MAD levels in the VD3 group compared to the baseline (*p* = 0.14, Table 2). Similarly, when comparing the intervention and placebo groups, no significant differences were detected in TAC (*p* = 0.81) and thiol levels (*p* = 0.28). These results indicate that the antioxidant effects of VD3 may require higher doses or longer intervention durations. Similarly, no significant differences were observed in HbA1c levels between the two groups at the end of the study (*p* = 0.70, Table 2). Although a slight decrease was noted in the intervention group, it did not reach statistical significance (*p* = 0.12). These findings suggest that while vitamin D3 supplementation may influence glycemic control, the observed effect might require a longer intervention or higher doses to achieve statistical significance. The effects of vitamin D3 supplementation varied over time between the intervention and placebo groups, a two‐way repeated measures ANOVA was performed. No significant interaction effects (Time × Group) were observed for BMI (*p* = 0.414), systolic BP (*p* = 0.473), diastolic BP (*p* = 0.668), HbA1c (*p* = 0.503), TAC (*p* = 0.811), MDA (*p* = 0.700), and thiol levels (*p* = 0.279), confirming that the trends over time were comparable between both groups (Table 2). The low effect sizes suggest that the vitamin D3 intervention did not induce substantial changes in these variables within the study duration.

### 3.4 Effect of VD3 on Gene Expression

**Figure 2:**
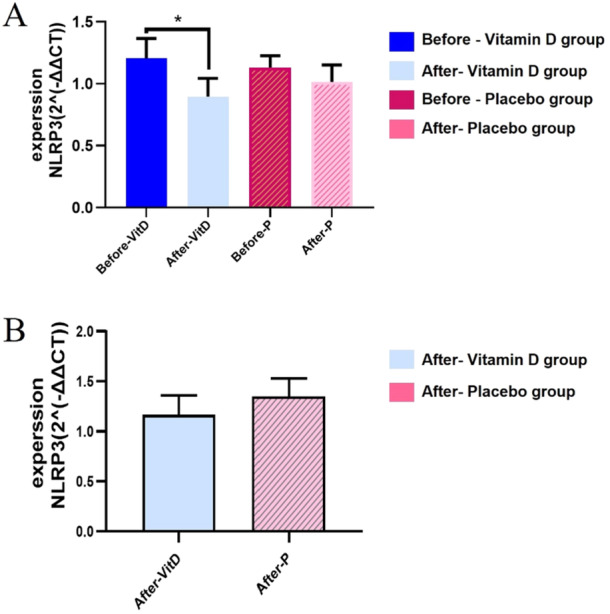
Shows changes in NLRP3 gene expression in the intervention groups. (A) Fold change of NLRP3 Compared with baseline in two the vitamin D3 group and the Placebo with type 2 diabetes. (B) Fold changes of NLRP3 in the endpoint (vitamin D3 group Compared with the Placebo); Data are presented as Mean ± SEM. *p < 0.05.

Figure 2 shows the changes in NRPL3 gene expression in the studied groups. Improvement's in VD3 status significantly decreased the expression of NRPL3 (*p* < 0.02). Nevertheless, the comparison of changes in the outcomes between the VD3 and placebo groups was not significant (*p* > 0.05).

The side effects of the drug dose were evaluated by a questionnaire, and no adverse events were noted in this respect.

## 4 Discussion

### 4.1 Main Findings

This study sought to examine the anti‐inflammatory and antioxidant effects of 25‐hydroxyvitamin D3 (25(OH)VD3) in diabetic patients with vitamin D3 deficiency over an 8‐week period. The intervention group exhibited a significant increase in 25(OH)VD3 levels compared to the placebo group. In contrast, a slight reduction in NLRP3 mRNA expression was observed in both groups, with a statistically significant decrease noted in the intervention group. These findings suggest that 25(OH)VD3 supplementation may modulate inflammatory and oxidative pathways in type 2 diabetes mellitus (T2DM). Evidence from animal and human studies indicates that vitamin D3 (VD3) may serve as a potential risk modifier for T2DM by reducing inflammation, oxidative stress, and cellular/tissue damage [17].

The results of the current study showed that the expression of the NLRP3 gene in the intervention group decreased significantly (Figure 2). In this regard, some studies have evaluated the effects of VD3 on the expression of inflammatory genes, which was in line with our results. For instance, Zhang et al (2017) evaluated the relationship between VD3 levels and the activity of the NLRP3 inflammasome in the pathogenesis of asthma in a model of obese mice. Obese rats with asthma showed significant increases in airway inflammation, pro‐inflammatory cytokine levels, and NLRP3 and IL‐1b mRNA expression. These results were obtained when both asthma and obese groups had lower 25(OH) VD3 levels. In addition, the amount of VD3 in obese asthma rats was lower than that of all groups [20]. Also, Tulk et al (2015) showed that VD3 indirectly decreases the expression of the NLRP3 gene [21]. Vitamin D can reduce NLRP3 gene expression through vitamin D receptor (VDR) dependent mechanisms [20, 21].

While our study demonstrated a significant reduction in NLRP3 expression with VD3 supplementation, no significant changes were observed in oxidative stress markers (MDA, TAC, thiol levels). These findings suggest that the antioxidant effects of VD3 might depend on factors such as dosage, intervention duration, or individual variability. Further studies are needed to investigate these parameters and their relationship with oxidative stress. The decrease in MDA levels and the increase in TAC and thiol groups in the VD3 group compared to the placebo group demonstrate the protective effects of VD3 in reducing oxidative stress and enhancing the antioxidant defense system. These results were consistent with previous studies [22, 23]. These effects can be linked to several molecular mechanisms associated with VD3. This vitamin, through binding to the vitamin D receptor (VDR), which is active in cells, especially in the nucleus, can stimulate the regulation of antioxidant genes [23]. This interaction leads to the activation of genes like glutathione peroxidase (GPX), superoxide dismutase (SOD), and catalase, all of which play roles in reducing oxidative stress and protecting cells from oxidative damage [24, 25]. VD3 can also indirectly help reduce the production of reactive oxygen species (ROS) by suppressing the NF‐κB signaling pathway, which is crucial in creating inflammatory responses and oxidative stress [26, 27]. The downregulation of this pathway can help decrease MDA levels and reduce damage caused by free radicals [27]. Additionally, VD3 may enhance the activity of thiols (SH‐groups), which are key molecules in antioxidant defense [28]. Thiols play a role in neutralizing free radicals and preventing damage to cellular molecules such as proteins, DNA, and lipids [29]. Furthermore, VD3 plays an important role in regulating intracellular calcium levels. Increased calcium in the cytoplasm can lead to the activation of enzymes such as calcium/calmodulin‐dependent protein kinase (CaMK) and calcium‐dependent phospholipases, which, in turn, contribute to the regulation of antioxidant pathways [30]. Therefore, VD3, through several molecular mechanisms including the suppression of inflammatory pathways, activation of antioxidant pathways [31], and regulation of intracellular calcium levels [32] helps reduce oxidative stress and enhance the body's antioxidant capacity. These effects may support the potential use of vitamin D3 as an adjunctive therapy in conditions associated with oxidative stress and inflammation.

### 4.2 Strength and Limitation

The strong point of our study lies in the study design, which is a double‐blinded randomized controlled trial. The participants in this study were homogeneous and matched for age and gender. 25(OH)VD3 was measured among the metabolites of VD3 because its half‐life in the bloodstream lasts for 15 days. Moreover, its level is regulated by parathyroid hormone, calcium, and phosphorus. Additionally, the 25(OH)VD3 level usually remains constant and decreases only during severe VD3 deficiency [33, 34]. Eventually, this study was conducted during winter to maintain low VD3 levels in the placebo group. On the other hand, the limitation of the study is that the participants were trained to keep their diet constant while they were not vigilantly watched during the study period. Furthermore, lack of detailed documentation on the types of medications the patients were taking at the time of the trial is noted. Certain medications, particularly metformin and insulin, which are widely used in the treatment of type 2 diabetes, have anti‐inflammatory properties and may influence markers related to inflammation and oxidative stress. We acknowledge this issue. However, since all participants in this study were on antidiabetic medications, and the majority had a history of diabetes for over 3 years, the potential confounding effect of these medications was considered uniform across the groups. This uniformity minimizes the risk of bias caused using these medications. Nevertheless, we recommend future studies to include a detailed analysis of the effects of specific medications on inflammatory and oxidative stress markers to further enhance the reliability of the findings.

## 5 Conclusion

Our findings indicate that vitamin D3 supplementation reduces NLRP3 gene expression in patients with T2DM, suggesting a potential role in modulating inflammation. However, no significant changes were observed in oxidative stress markers, indicating that the antioxidant effects of vitamin D3 may depend on factors such as dosage, intervention duration, or individual variability. Further large‐scale and long‐term studies are needed to clarify the precise mechanisms and clinical implications of vitamin D3 supplementation in managing inflammation and oxidative stress in T2DM patients.

## Author Contributions

**Shadi Behshad:** conceptualization, investigation, writing – original draft, writing – review and editing, validation. **Yaser Mohammadi:** investigation, writing – original draft, writing – review and editing, validation, formal analysis. **Gholamreza Anani Sarab:** methodology, writing – review and editing, software, conceptualization, validation. **Mohammad Malekaneh:** conceptualization, writing – review and editing, visualization, validation, methodology, data curation, supervision. **Azam Rezaei Farimani:** conceptualization, investigation, funding acquisition, writing – review and editing, validation, methodology, software, formal analysis, supervision, project administration.

## Ethics Statement

This study was approved by the Ethics Committee of Birjand University of Medical Sciences with Ethic's ID: IR.BUMS.REC.1398.140, also registered on the IRCT website with the code IRCT20200616047795N1.

## Conflicts of Interest

The authors declare no conflicts of interest and are responsible for the content and writing of the paper.

## Transparency Statement

The lead author Mohammad Malekaneh, Azam Rezaei Farimani affirms that this manuscript is an honest, accurate, and transparent account of the study being reported; that no important aspects of the study have been omitted; and that any discrepancies from the study as planned (and, if relevant, registered) have been explained.

## Data Availability

The data that support the findings of this study are available from the corresponding author upon reasonable request.
